# The Genome and Transcriptome Analysis of the *Vigna mungo* Chloroplast

**DOI:** 10.3390/plants9091247

**Published:** 2020-09-21

**Authors:** Wanapinun Nawae, Chutintorn Yundaeng, Chaiwat Naktang, Wasitthee Kongkachana, Thippawan Yoocha, Chutima Sonthirod, Nattapol Narong, Prakit Somta, Kularb Laosatit, Sithichoke Tangphatsornruang, Wirulda Pootakham

**Affiliations:** 1 National Omics Center (NOC), National Science and Technology Development Agency, 111 Thailand Science Park, Khlong Nueng, Khlong Luang, Pathum Thani 12120, Thailand; wanapinun.naw@nstda.or.th (W.N.); chutintorn.yun@nstda.or.th (C.Y.); chaiwat.nak@nstda.or.th (C.N.); wasitthee.kon@nstda.or.th (W.K.); thippawan.yoo@nstda.or.th (T.Y.); chutima.son@nstda.or.th (C.S.); nattapol.nar@nstda.or.th (N.N.); sithichoke.tan@nstda.or.th (S.T.); 2 Department of Agronomy, Faculty of Agriculture at Kamphaeng Saen, Kasetsart University, Nakhon Pathom 73140, Thailand; agrpks@ku.ac.th (P.S.); fagrkal@ku.ac.th (K.L.)

**Keywords:** chloroplast genome, chloroplast transcriptome, legume, *Vigna mungo*, comparative analysis, positive selection, RNA editing, polycistronic transcription

## Abstract

*Vigna mungo* is cultivated in approximately 5 million hectares worldwide. The chloroplast genome of this species has not been previously reported. In this study, we sequenced the genome and transcriptome of the *V. mungo* chloroplast. We identified many positively selected genes in the photosynthetic pathway (e.g., *rbcL*, *ndhF*, and *atpF*) and RNA polymerase genes (e.g., *rpoC2*) from the comparison of the chloroplast genome of *V. mungo*, temperate legume species, and tropical legume species. Our transcriptome data from PacBio isoform sequencing showed that the 51-kb DNA inversion could affect the transcriptional regulation of *accD* polycistronic. Using Illumina deep RNA sequencing, we found RNA editing of *clpP* in the leaf, shoot, flower, fruit, and root tissues of *V. mungo*. We also found three G-to-A RNA editing events that change guanine to adenine in the transcripts transcribed from the adenine-rich regions of the *ycf4* gene. The edited guanine bases were found particularly in the chloroplast genome of the *Vigna* species. These G-to-A RNA editing events were likely to provide a mechanism for correcting DNA base mutations. The *V. mungo* chloroplast genome sequence and the analysis results obtained in this study can apply to phylogenetic studies and chloroplast genome engineering.

## 1. Introduction

*Vigna mungo* (L.) Hepper or black gram is a diploid plant with 2*n* = 2*x* = 22 chromosomes. It belongs to the family Leguminosae, subfamily Papilionoideae, clade Millettioid [1], and is a tropical legume crop species that is cultivated in Asia, Africa, and America [2]. Black gram is an economically important *Vigna* species, which provides high-protein food [3].

Chloroplast is an essential organelle that harbors about 120–130 genes in its own genome [4]. The genera *Phaseolus* [5], *Glycine* [6], *Vigna* [7], and *Cajanus* [8] are examples of the tribe Phaseoleae species that have their complete chloroplast genome sequence reported. Structural variations including inverted repeat region (IR) expansion or contraction, genome rearrangement, loss of gene or intron, and pseudogenes among these legumes are described [9]. The chloroplast genome of *Vigna radiata* has been sequenced [7,10], where Lin et al., 2015 additionally used RNA-seq data to identify RNA editing events [10]. Many recent chloroplast genome studies focus their analysis on identifying positively selected genes, RNA editing events, and polycistronic transcription units [11,12,13]. The identification of the positively selected genes is important for evolutionary studies because these genes had a fixation of advantageous point mutations (positively selected sites) as an adaptation to the selective force (positive selection) from different ecological conditions [14,15]. The non-synonymous (Ka) and the synonymous (Ks) nucleotide substitution rates and the Ka/Ks ratio are commonly used to detect positive selection or adaptive evolution events [13]. For example, the adaptive evolution of chloroplast genes as identified by Ka/Ks ratios was reported to be responsible for the adaptation of rice species to diverse ecological habitats related to sunlight preferences [13]. Detailed sequences of genes and intergenic regions from the chloroplast genome are often used to study the phylogenetic relationship of plant species [16,17]. Recently, the phylogenetic tree generated from the sequence of 80 plastid genes from 2,514 plant species was used to estimate the origin and divergence time of angiosperms [18]. Moreover, chloroplast genomes are the targets for genetic engineering in the agricultural, pharmaceutical, and medical applications [19,20,21]. For example, the engineering of chloroplast genomes could increase the tolerance of plants to high temperature [22] and salt stress [23] and to confer insecticidal activity to the transgenic plants [24]. Pharmaceutically, the chloroplast genome engineering enabled the low-cost production of, for example, polio vaccine [25], interferon-α2b [26], and proinsulin [27]. Therefore, more information on the sequence, structure, and transcription of the chloroplast genomes enable us to better understand plant evolution and to effectively use this organelle in a broader biotechnological application.

In this study, we sequenced the chloroplast genome of *V. mungo*. We identified positively selected genes in the *V. mungo* chloroplast genome by performing comparative analysis with chloroplast genomes from the related legume species that preferred different climates to grow. We additionally used PacBio isoform sequencing (Iso-seq) reads to show polycistronic transcription units and used Illumina short reads to identify RNA edited sites in different *V. mungo* tissues.

## 2. Results and Discussion

### 2.1. General Features of Vigna Mungo Chloroplast Genome

**Figure 1:**
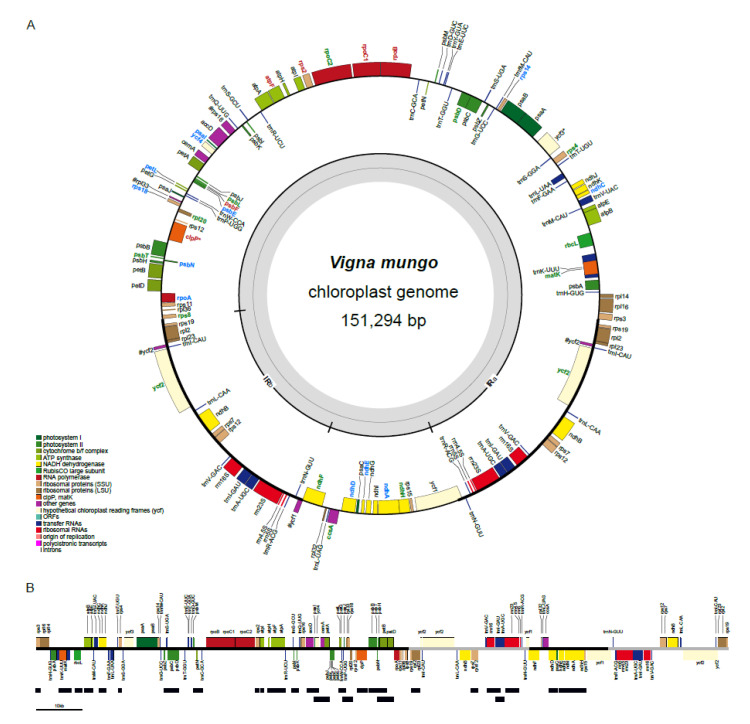
Structure and expression of the chloroplast genome of *Vigna mungo*. (**A**) The structure of the *V. mungo* chloroplast genome is shown. The genes inside the circle are transcribed clockwise, and genes outside are transcribed counter-clockwise. Genes from different functional groups are shown in different colors. Genes with either RNA editing or positive selection are labeled with blue or green text colors, respectively. Genes with both RNA editing and positive selection are labeled with red text colors. (**B**) Polycistronic transcription units (black bars) are shown relative to the position of genes on the *V. mungo* chloroplast genome, which is visualized in a linear form for simplicity.

We obtained 4.4 Gb from 15 million Illumina paired-end (PE) reads for chloroplast genome assembly. The raw reads were deposited to the National Center for Biotechnology Information (NCBI) database under the BioProject accession number of PRJNA623719. The assembled chloroplast genome of *V. mungo* was 151,294 base pairs (bp) long (Figure 1A). We have deposited the *V. mungo* chloroplast genome to the GenBank Nucleotide Database with the accession number MT418597. The average depth coverage across the genome was 61.98× (Figure S1). The genome has a circular quadripartite structure with one large single copy (LSC; 80,984 bp), one small single copy (SSC; 17,448 bp), and two inverted repeat regions (IRa and IRb; 26,431 × 2 bp). The IRa and IRb were repeated sequences appearing in the inverted direction in the circular structure of the chloroplast genome. The overall GC content of the chloroplast genome is 35.24%. The GC content of the LSC, SSC, and each of IR regions were 32.59%, 28.54%, and 41.52%, respectively, which were consistent with that of the chloroplast genome of *V. radiata* (mungbean) [7]. The *V. mungo* chloroplast genome has 108 genes including 75 protein-coding genes, 29 tRNA genes, and four rRNA genes (Table 1). The LSC region contained 57 protein-coding genes and 21 tRNA genes, while the SSC region had 12 protein-coding genes and one tRNA gene. In each of the IR regions, there are six protein-coding genes, four rRNA genes, and seven tRNA genes. The gene density was slightly higher in the LSC region (963 genes/Mb) than in the SSC (745 genes/Mb) and the IR regions (643 genes/Mb). Nine protein-coding genes and five tRNA genes had one intron. *ycf3* and *clpP* were two genes that had two introns. We found *^ψ^rpl33* and*^ψ^rps16* pseudogenes in the LSC region and *^ψ^ycf1* pseudogene, which spanned the IRb/SSC (JSB) boundary. The *^ψ^rpl33* was shown be specific to Phaseolinae chloroplast genomes, while *^ψ^rps16* was found to be absent in the *Ceratonia siliqua* and *Glycine max* chloroplast genomes [28].

### 2.2. Comparative Chloroplast Genome Analysis

We compared the *V. mungo* chloroplast genome sequenced in this study with 11 other Leguminosae chloroplast genomes that were downloaded from the GenBank database. Five of these species were *V. radiata*, *Vigna angularis*, *Vigna unguiculata*, and *Phaseolus vulgaris*, and *G. max* was from the tribe Phaseoleae. The first four species are member of the subtribe Phaseolinae, while *G. max* is from the subtribe Glycininae. The other four species were *Glycyrrhiza glabra*, *Cicer arietinum*, *Pisum sativum*, and *Medicago sativa* from the IR-lacking clade. Two other species were *Arachis hypogaea* from the tribe Dalbergieae and *Ceratonia siliqua* from the subfamily Caesalpinioideae.

**Figure 2:**
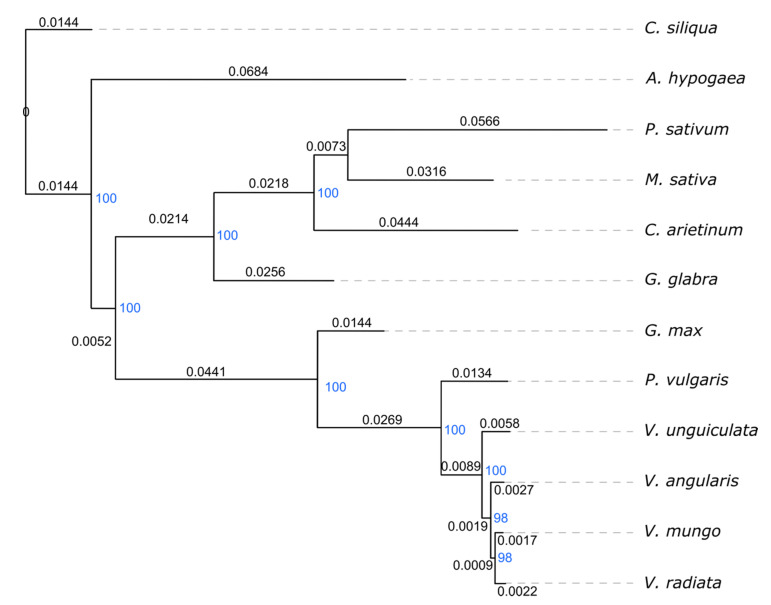
Phylogenetic relationship of the analyzed legumes. The phylogenetic tree was calculated from orthologous genes from the chloroplast genome of *V. mungo* and other eleven Leguminosae species. *Ceratonia siliqua* was used as an outgroup in the phylogenetic tree calculation. Bootstrap values are shown in blue color, and branch lengths are shown in black color.

We generated phylogenetic tree for these species using 69 orthologous genes (Table S1). As a result, the species from the tribe Phaseoleae formed a sister clade to the species from the IR-lacking clade (Figure 2). These two clades shared commons ancestors with *Arachis* and *Ceratonia*, respectively. *V. mungo* formed a monophyletic group with *V. radiata* at the deepest branch of the Phaseolinae clade, indicating a close evolutionary relationship between these two species. We also calculated the phylogenetic tree of these species with 57 other species to confirm the placement of these Fabales species in relative to other diverse species. This calculation used 38 orthologous protein sequences from the chloroplast genomes. In resulted phylogenetic tree (Figure S2), the Fabales species formed a sister clade to the Rosales, Fagales, and Cucurbitales clades, which was consistent with the tree that was calculated from 80 genes from 2881 chloroplast genomes [18].

**Figure 3:**
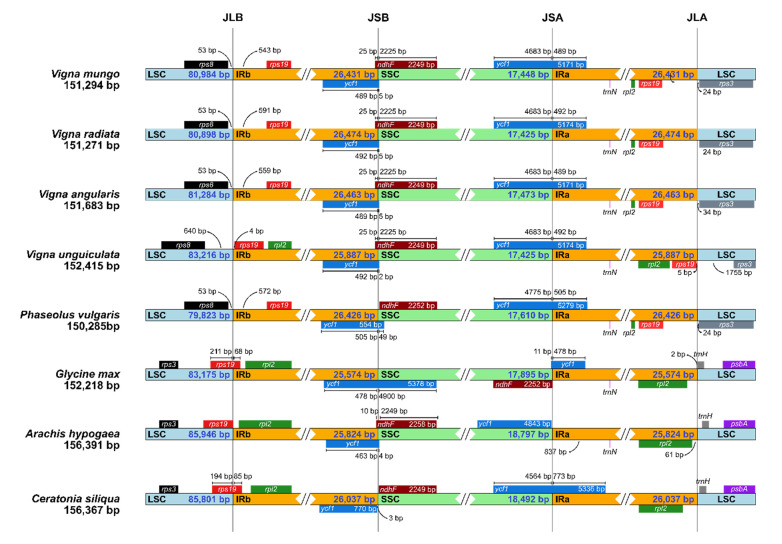
IR expansion/contraction. Large single copy (LSC), small single copy (SSC), and IR regions among eight chloroplast genomes that have two IR region are compared. The numbers inside the LSC (cyan), SSC (green), and IR (orange) regions show the length of the corresponding regions. The numbers outside the regions show the distances relative to the region boundaries.

As a structural analysis, the IR expansion/contraction of the *V. mungo* chloroplast genome were examined. During the evolution of land plant, the IR expansion caused the movement of the entire or a portion of genes from the SC regions into the IR regions and vice versa for the IR contraction [29]. In this study, we found that *V. mungo* and other Phaseolinae species had two copies of *rps19* inside their IR regions (Figure 3). In contrast, *G. max*, *A. hypogaea*, and *C. siliqua* had one copy of *rps19*, although they had two IR regions (Table S1). *rps19* of these three species spanned the LSC/IRb boundaries (Figure 3). In addition, the length of the IR regions in the *V. mungo* chloroplast genome was 857, 394, and 607 bp longer than the IR regions from the *G. max*, *A. hypogaea*, and *C. siliqua* chloroplast genomes, respectively. Altogether, our results indicated that the IR regions were expanded in the chloroplast genome of *V. mungo*.

**Figure 4:**
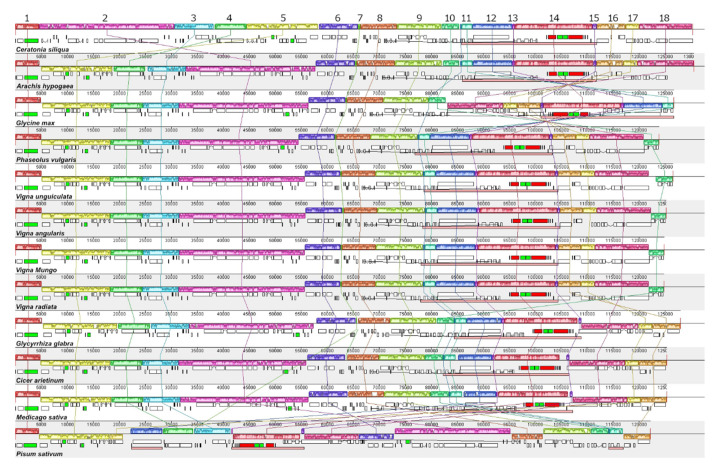
Chloroplast genome rearrangement. Eighteen locally collinear blocks (LCBs) are shown with different colors. Each LCB is numbered according to their order in the *C. siliqua* chloroplast genome. The LCB numbers are shown on top of the LCBs of the *C. siliqua* chloroplast genome. The LCBs with conserved sequences among the compared chloroplast genomes are represented in the same color. For the chloroplast genomes that have two IR regions, only the IRb region is considered in the alignment. The IR region of each genome is marked with pink bar. The line graph inside each LCB shows the sequence similarity level. Corresponding LCBs from different species are connected by lines that have the same color as the LCB that they connect to.

We used Mauve aligner [30] to align the chloroplast genome of the species in the Fabales clade to investigate genome rearrangement. The alignment calculated the locally collinear blocks (LCBs), which presented the high similarity conserved regions among the compared genomes. For Mauve alignment, we removed the IRa sequence from the analyzed chloroplast genomes that had two IR regions. This modification allowed the program to calculate the LCBs for the repetitive sequence of the IR regions. We obtained 18 LCBs from the alignment using the *C. siliqua* chloroplast genome as a reference sequence (Figure 4). We identified the rearrangement of the *V. mungo* chloroplast genome based on the relative position of these LCBs. We found an inversion of the DNA segment covering from LCB2 to LCB5 in the chloroplast genome of *V. mungo* and all other Faboideae species, except for *P. sativum* (Figure 4). This inversion coincided with the 51-kb inversion that was reported to occur early in the diversification of papilionoid legumes [31]. Compared to the *A. hypogaea* chloroplast genome, the *G. max* had the inversion of the DNA segment covering from LCB11 to LCB18, which covered the SSC region and the IR regions. In the chloroplast genome of *V. mungo* and other Phaseolinae species, this DNA segment was reinverted to the same arrangement as the chloroplast genome of *A. hypogaea* (Figure 4). Additional DNA segment that changed it location with this reinversion was LCB10, which was a part of LSC regions. The inversion of this LCB10-to-LCB18 DNA segment correspond to the 78-kb inversion reported in other Phaseolinae species [5,7]. These results supported that the 51-kb inversion and the 78-kb inversion were characteristics of the chloroplast genomes from the subtribe Phaseolinae.

### 2.3. Positively Selected Genes

In this study, we calculated the non-synonymous (Ka) to synonymous (Ks) substitution ratio (Ka/Ks) for each of the 60 protein-coding genes shared by the analyzed legumes. The Ka/Ks ratio showed the strength and mode of natural selection on the protein-coding genes [32]. The Ka/Ks > 1 indicated that the corresponding genes experienced positive selection while the genes experiencing neutral or purifying (negative) selection were indicated by Ka/Ks = 1 or Ka/Ks < 1, respectively [32,33]. The average Ka/Ks ratio of the 61 protein-coding genes of the *V. mungo* chloroplast genome was 1.77. We found *V. mungo* chloroplast genome had 19 positively selected genes (Ka/Ks > 1 [34,35]) including eight photosynthetic genes (42%), four ribosome genes (21%), three RNA polymerase genes (16%), and four other genes (21%) (Figure 1A and Table 2). We found that *rbcL* and *ycf2* had the highest number of positively selected sites (11 sites), followed by *ndhF* (7 sites), *atpF* and *rps2* (5 sites), *ndhH* (4 sites), *matK*, *psbT* and *rpoC1* (3 sites), and other genes (<2 sites) (Table 2).

Amino acid changes due to the selection pressure can drive evolution within a specific taxonomic lineage [36]. Positive selection is a process, in which advantageous mutations increase fitness of plants to the ecological habitats [37]. Leguminosae consists of 19,500 species with a worldwide distribution [38], which can be grouped, based on growing seasons, into cool-season food legumes and warm-season food legumes [39]. For the legumes analyzed in this study, *P. sativum* (dry pea) and *C. arietinum* (chickpea) are members of the cool-season food legumes, while *V. radiata* (mungbean), *V. mungo* (black gram), and *P. vulgaris* (common bean) grow well under hot and humid conditions [39]. We used legume species that prefer different climatic conditions for calculating the Ka/Ks ratio by hypothesizing that these species experienced different ecologically selective pressures. Many positively selected genes, e.g., *rbcL*, *matK*, *ndhF*, *atpF*, and *rpoC2*, in the *V. mungo* chloroplast genome were found to be involved in ecological adaptations in other plant species. For example, an adaptive evolution in the *rbcL* gene is linked with photosynthetic performance under temperature, drought and carbon dioxide concentration variations [40]. Rubisco had a small carboxylase turnover rate and low CO_2_ affinity, which limits the rate of carbon assimilation and net photosynthesis [41]. The improvable activities of Rubisco might explain the *rbcL* positive selection that was widespread in many land plants [41]. The positive selection of *matK* was also identified in many plants suggesting that this gene experienced different ecological selective pressures [19,42]. In the genus *Citrus*, positive selection of *matK* and *ndhF* was considered to play roles in the adaptation of Australian species to hot and dry climate [19]. In addition, positive selection of *ndhF* was believed to be able to delay drought-induced senescence in *Haberlea rhodopensis* [43]. The gene sequence comparisons between deciduous *Quercus* species and evergreen *Quercus* species revealed that the positive selection of *atpF* and *rpoC2* played a role in the adaptation of the deciduous *Quercus* species to the winter or the dry season [44]. The positive selections identified in this study were likely related to the different climatic preferences among the analyzed legume species.

In addition to climatic conditions, some legumes prefer different light spectrums as shown by a study indicating that the red light significantly suppressed pod growth in soybean, but promoted the growth in cowpea [45]. A study with 22 chloroplast genomes from the genus *Oryza* that were grouped into shade-tolerant and sun-loving rice species showed a correlation between the positive selection of photosynthetic genes, e.g., *rbcL*, *ndh*, and *psb*, and the adaptation of rice species to different sunlight levels [13]. The positive selection of these genes was also observed in our Ka/Ks analysis, which included soybean and cowpea (Table 2). Light spectrums might be other possible selection pressure exerted to the chloroplast gene of the analyzed legumes.

### 2.4. Polycistronic Transcription Units

The transcriptional regulation of chloroplast genes has characteristics found in both prokaryotes and eukaryotes [46]. Plastid genes are co-transcribed into polycistronic transcription units, which resemble operons in bacteria [46,47]. Primary polycistronic transcripts then undergo post-transcriptional modifications (for example, RNA editing), which is a characteristic of eukaryotes [46,48]. In this study, we investigated both polycistronic transcription units and RNA editing events in the *V. mungo* chloroplast genome.

This study used PacBio Isoform Sequencing (Iso-Seq) to identify polycistronic transcription units of the *V. mungo* chloroplast genome. We retrieved 10 monocistronic units, seven dicistronic units, and 11 polycistronic units (Figure 1B). We found three overlapping pairs of polycistronic units (*accD*-*psaI*, *petL*-*psaJ*, and *psbB*-*psbN*). The degradation of a long polycistronic unit into smaller oligocistronic units could result from intercistronic processing activities [49]. For example, in *Arabidopsis thaliana*, the binding of HCF152 to the intergenic region between *psbH* and *petB* was involved in the digestion of the *psbB*-*psbT*-*psbH*-*petB*-*petD* polycistronic units by exonucleases to *psbB*-*psbT*-*psbH* tricistronic units and *petB*-*petD* dicistronic units [50]. In this study, we found coexistence of *psbB*-*psbT*-*psbN*-*psbH*-*petB* and *psbN*-*psbH*-*petB*-*petD* polycistronic units, suggesting that there may be binding sites in the intergenic region between *psbT* and *psbN* for a protein with a similar function to HCF152 in the *V. mungo* chloroplast genome.

The Iso-Seq data also provided evidence that the *accD* polycistronic transcripts in *V. mungo* were transcribed from the *rps16* promoter, locating further upstream of *accD*, due to a 51-kb inversion between *accD*/*rps16* and *rbcL*/*trnK*-*UUU*. We observed that the promoter sequence of *accD* in *V. mungo* is truncated and lacks the GAA-box compared to that of *C. siliqua*, a legume without the 51-kb inversion. These results suggested that the 51-kb inversion affect transcriptional regulation of the *accD* polycistronic transcripts.

The iso-seq reads also allow for the detection of alternative splicing events. There are two genes (*ycf3* and *clpP*) with two introns in the *V. mungo* chloroplast genome. We have mapped our iso-seq reads to the genome. However, the results showed no evidence of an alternative splicing event in either of these genes.

### 2.5. RNA Editing

In this study, we identified 34 RNA editing events in 18 plastid genes from leaf, shoot, flower, fruit, and root tissues of *V. mungo* using RNA-seq data (Figure 1A and Table 3). Ninety-one percent of these RNA editing events were C-to-U editing, and the remaining were G-to-A editing. RNA editing occurred at different efficiency levels in different tissues [51,52]. A majority of editing events in *V. mungo* were found in leaves, which was consistent with a study in *A. thaliana* [52]. The number of RNAseq reads from leaves that were uniquely mapped to the chloroplast genome (44,607 reads) was higher than the number of reads from other tissues (16,631 reads from shoots, 17,501 reads from flowers, 4356 reads from fruits, and 8334 reads from roots). The results suggested different expression levels in different tissues and might explain the highest number of the edited sites in leaves compared to other tissues of this study. We also found that different tissues had different RNA edited events. It was only the C-to-U editing of *clpP* (*clpP*-556) that was commonly found in all five tissues (Table 3). Our Ka/Ks analysis also showed that *clpP* was one of the positively selected gene of the *V. mungo* chloroplast genome (Table 2). *clpP* has been reported to express in chloroplasts and non-photosynthetic plastids and has been shown to be involved in chloroplast development and cell survival [53]. The disruption of *clb19*, which was involved in *clpP* editing, impaired chloroplast development, caused yellow phenotype, and increased early seedling lethality rate [54]. *clb19* and the corresponding *clpP*-559 editing in the *A. thaliana* chloroplast genome (equivalent to the *clpP*-556 editing in the *V. mungo* chloroplast genome) are absent from core asterids and Poaceae but are retained in most of rosids [55]. *Vicia faba* and *C. arietinum* are two legumes that lost CLB19 and *clpP*-559 editing [55]. Our results, together with the results from these studies, suggested that the sequence and the RNA editing of *clpP* can be used as a good marker for studying phylogenetics.

RNA-seq data allow for the detection of edited sites with low editing efficiency [56,57]. We found three G-to-A editing events in *ycf4* from leaves (*ycf4*-20, *ycf4*-309, and *ycf4*-316), although they had low editing efficiency. G-to-A editing has also been reported in *ndhF*, *rpoC2*, and *ycf1* genes of the *A. thaliana* [52] and *Betula platyphylla* [58]. In the *V. mungo* chloroplast genome, we found the edited G bases in two A-rich regions (Figure S3), where the sequences were translated to long lysine chains. The *ycf4*-20 and *ycf4*-316 editing resulted in an amino acid change from arginine and glutamic acid to lysine, respectively (Table 3). The *ycf4*-309 editing event was a synonymous editing, which did not change lysine amino acid in the protein sequence. The conversion of G to A in these A-rich regions might facilitate three-dimensional folding or interaction of the protein products translated from the edited transcripts of *ycf4*. All analyzed *Vigna* species have these three edited G bases in *ycf4*, but the *P. vulgaris* chloroplast genome has only one G base homologous to *ycf4*-20 (Figure S3). The edited G bases were likely specific to the chloroplast genome of *Vigna* species and might have resulted from mutation events. The observed G-to-A editing events in the *V. mungo ycf4* transcripts support the idea that RNA editing is a mechanism to correct mutations in the genomic coding sequences, which accumulated during evolution process [57,59].

## 3. Materials and Methods

### 3.1. DNA and RNA Extraction

Black gram samples were obtained from Kasetsart University (Nakhon Pathom, Thailand). Frozen tissues (CN80 accession) were homogenized, and DNA was extracted using QIAGEN Genomic-tip 100/G based on the manufacturer’s protocol (Qiagen, Hilden, Germany). We assessed DNA integrity with the Pippin Pulse Electrophoresis System (Sage Science, Beverly, MA, USA). Total RNA was isolated from leaf, root, stem, flower, and three-week-old pod using the CTAB buffer (2% CTAB, 1.4 M 91 NaCl, 2% PVP, 20 mM EDTA pH 8.0, 100 mM Tris-HCl pH 8.0, 0.4% SDS). RNA was extracted from the aqueous phase three times using 25:24:1 phenol:chloroform:isoamylalcohol. Poly(A) enrichment with Dynabeads mRNA Purification Kit (ThermoFisher Scientific, Waltham, MA, USA) was used to get mRNAs.

### 3.2. Preparation of DNA and RNA Libraries and Sequencing

For DNA sequencing, we prepared sequencing library from a total of 1.25 ng of high molecular weight DNA using the Chromium Genome Library Kit & Gel Bead Kit v2, the Chromium Genome Chip Kit v2, and the Chromium i7 Multiplex Kit following the manufacturer’s instructions (10X Genomics, Pleasanton, CA, USA). We used a single lane of Illumina HiSeq X Ten (2 × 150 bp paired-end reads) to sequenced the library.

For RNA sequencing, RNA integrity was assessed with a Fragment Analyzer System (Agilent, Santa Clara, CA, USA). To obtain short-read RNA sequences, six RNA libraries (one for each tissue type) were prepared according to the protocol reported in Pootakham et al., 2018. Briefly, 200 ng of poly(A) mRNA was used to construct a library using the Ion Total RNA Sequencing Kit (ThermoFisher Scientific, Waltham, MA, USA). The libraries were sequenced on the Ion S5 XL using the Ion 540 TM chip (ThermoFisher Scientific, Waltham, MA, USA). For RNA isoform sequencing, two PacBio Iso-seq libraries were prepared following protocols described in [60] using the SMARTer PCR cDNA Synthesis Kit (Clontech, Mountain View, CA, USA) and size-selected using the BluePippin Size Selection System (Sage Science, Beverly, MA, USA) into 1–2 kb, 2–3 kb, and 3–6 kb bins. The Iso-seq sequencing was performed on the PacBio RSII sequencing system (Pacific Biosciences, Menlo Park, USA, outsourced to NovogenAIT, Singapore) using P6-C4 polymerase and chemistry and 360 min movie times according to the manufacturer’s protocol.

### 3.3. Genome Assembly and Annotation

The raw reads were trimmed and filtered for high quality reads by fastp program with default parameters [61]. We used the GetOrganelle pipeline [62] to de novo assemble the genome sequence. In the pipeline, we set word size ratio to 0.4 for extracting chloroplast reads from total DNA reads and used a combined k-mer of 95,105,125 together with k-mer gradient for de novo assembly with SPAdes [63]. We ran the “evaluate_assembly_using_mapping.py” script from the GetOrganelle software package to evaluate depth of coverage across the assembled chloroplast genome. We used CPGAVAS2 [64], GeSeq [65], and Geneious [66] for chloroplast genome annotation. The annotated chloroplast genome was visualized with OGDRAW [67].

### 3.4. Comparative Analysis of V. mungo Chloroplast Genome

We used Mauve aligner software [30] with default parameters to align the *V. mungo* chloroplast genome with chloroplast genome of *V. radiata* (NC_013843.1), *V. angularis* (NC_021091.1), *V. unguiculata* (NC_018051.1), *P. vulgaris* (NC_009259.1), *G. max* (NC_007942.1), *G. glabra* (NC_024038.1), *C. arietinum* (NC_011163.1)*, P. sativum* (NC_014057.1)*, M. sativa* (NC_042841.1), *A. hypogaea* (NC_037358.1)*,* and *C. siliqua* (NC_026678.1). The sequence of the *C. siliqua* chloroplast genome was used as a reference for visualizing the LCB relative locations. To analyze IR expansion/contraction, IRscope [68] was used to compare size of the LSC region, the SSC region, and IR region among analyzed species.

### 3.5. Positive Selection

The codon-based alignment of the protein-coding gene sequences were conducted with the integrated MUSCLE alignment program of MEGA-CC software [69,70]. The positive selected genes and sites were identified with CODEML program of PAML 4 package [71] via the EasyCodeML interface [72]. The likelihood ratio tests (LRT) with a *p*-value cutoff of 0.05 was used to compared between the fitness of the alignment data to the M8 model (with the Ka/Ks > 1 parameter) and the M7 model (without the Ka/Ks > 1 parameter). Thereby, the identified positively selected genes had a high probability to experience positive selection than neutral selection.

### 3.6. RNA Editing Sites

We mapped RNA short reads from the Illumina deep RNA sequencing to the *V. mungo* chloroplast genome with Bowtie 2 software [73]. RNA editing sites were then identified with REDItools software [74]. We selected sites that had at least 15 RNA support reads and the frequency of the corresponding polymorphism at the DNA level (from DNA read mapping) lower than 0.01.

### 3.7. Polycistronic Analysis

To find genes on a polycistronic transcription unit, we aligned PacBio Iso-seq reads to the *V. mungo* chloroplast genome using BLASTN [75] with a 10^−3^ E-value cutoff. We selected the Iso-seq reads that had a 100% coverage and >95% identity alignment with the chloroplast genome sequence.

## 4. Conclusions

In this study, we sequenced the genome and transcriptome of the *V. mungo* chloroplast. The sequence and structure of the assembled chloroplast genome was consistent with the chloroplast genome of the closely related species of *V. mungo*. Our comparisons between the chloroplast genome of *V. mungo* and other legume species that grew in different habitats revealed many positively selected genes. Our RNA sequencing results showed that the 51-kb DNA inversion conserved among Papilionoideae legume species could affect the transcriptional regulation of the *accD* polycistronic transcription. Finally, we found RNA editing events that change guanine to adenine in the RNA molecules that were transcribed from the adenine-rich regions of the *ycf4* gene in the *V. mungo* chloroplast genome. The chloroplast genome sequence and the knowledge gained from this study can be used for plant phylogenetic studies and for the engineering of the chloroplast genome of *V. mungo* and other related legume species.

## Figures and Tables

**Table 1:** Annotated genes of the *V. mungo* chloroplast genome.

Category of Genes	Group of Genes	Name of Genes
Genes for photosynthesis	Subunits of photosystem II	psbA, psbB, psbC, psbD, psbE, psbF, psbH, psbI, psbJ, psbK, psbL, psbM, psbN, psbT, psbZ, ycf3 **
	Subunits of photosystem I	psaA, psaB, psaC, psaI, psaJ
	Subunits of NADH-dehydrogenase	ndhA *, ndhB *, ndhC, ndh, ndhE, ndhF, ndhG, ndhH, ndhI, ndhJ, ndhK
	Subunits of cytochrome b/f complex	petA, petB *, petD *, petG, petL, petN
	Subunits of ATP synthase	atpA, atpB, atpE, atpF *, atpH, atpI
	Subunit of rubisco	rbcL
Self-replication	Large subunit of ribosome	rpl14, rpl16 *, rpl2, rpl20, rpl23, rpl32, rpl36, rpl33^ψ^
	DNA dependent RNA polymerase	rpoA, rpoB, rpoC1 *, rpoC2
	Small subunit of ribosome	rps11, rps12 *, rps14, rps15, rps16 *^,ψ^, rps18, rps19, rps2, rps3, rps4, rps7, rps8
Other genes	Subunit of Acetyl-CoA-carboxylase	accD
	c-type cytochrom synthesis gene	ccsA
	Envelop membrane protein	cemA
	Protease	clpP **
	Maturase	matK
Ribosomal RNAs		rrn23S, rrn4.5S, rrn16S, rrn5S
Transfer RNAs	Ala	TrnA-UGC *
	Arg	trnR-ACG, trnR-UCU
	Asn	trnN-GUU
	Asp	trnD-GUC
	Cys	trnC-GCA
	Gln	trnQ-UUG
	Glu	trnE-UUC
	Gly	trnG-UCC
	His	trnH-GUG
	Ile	trnI-CAU, trnI-GAU *
	Leu	trnL-CAA, trnL-UAA *, trnL-UAG
	Lys	trnK-UUU *
	Met	trnM-CAU, trnfM-CAU
	Phe	trnF-GAA
	Pro	trnP-UGG
	Ser	trnS-GCU, trnS-GGA, trnS-UGA
	Thr	trnT-GGU, trnT-UGU
	Trp	trnW-CCA
	Tyr	TrnY-GUA
	Val	trnV-GAC, trnV-UAC *
Unkown	Conserved open reading frames	ycf1, ycf1 ^ψ^, ycf2, ycf4

* gene with one intron, ** gene with two introns, ^ψ^ pseudogene.

**Table 2:** Positively selected genes and sites in the *V. mungo* chloroplast genome.

Gene	Function	Ka/Ks of Gene	LRTs (2ΔLnL)	LRT *p*-Value	Selective Site	Pr(Ka/Ks > 1)	Ka/Ks of Site
atpF	Photosynthesis	1.94	0.39	0.02	39R	0.967 *	1.884
					62N	0.996 **	1.931
					79T	0.971 *	1.89
					83L	0.952 *	1.86
					167M	0.984 *	1.912
ccsA	Other genes	2.40	0.64	0.00	94Q	0.982 *	2.048
clpP	Other genes	2.87	0.83	0.04	12I	0.983 *	2.836
matK	Other genes	2.82	0.07	0.00	80V	0.956 *	2.725
					493Y	0.988 *	3.08
					494L	0.989 *	3.083
ndhF	Photosynthesis	1.55	0.55	0.00	64K	0.989 *	1.54
					289K	0.995 **	1.546
					507I	0.993 **	1.544
					616L	0.965 *	1.512
					638L	0.989 *	1.54
					740N	0.951 *	1.497
					741K	0.951 *	1.496
ndhH	Photosynthesis	1.03	0.15	0.00	3I	0.955 *	1.502
					176S	0.994 **	1.026
					269I	0.989 *	1.021
					294C	0.986 *	1.019
psbD	Photosynthesis	2.17	0.25	0.01	122G	1.000 **	2.168
psbE	Photosynthesis	3.94	0.17	0.05	59N	0.999 **	3.937
psbL	Photosynthesis	5.27	0.14	0.00	1M	1.000 **	5.267
psbT	Photosynthesis	1.71	0.01	0.00	28K	1.000 **	1.711
					33K	1.000 **	1.711
					34V	0.952 *	3.873
rbcL	Photosynthesis	1.64	0.16	0.00	28D	0.985 *	1.613
					86H	1.000 **	3.108
					95S	0.993 **	1.624
					97F	0.999 **	1.633
					142T	0.999 **	3.106
					228S	0.958 *	3.001
					251M	0.999 **	1.634
					375I	0.990 *	1.619
					449S	0.997 **	3.102
					470E	0.999 **	1.634
					475I	0.998 **	3.105
rpl20	subunit of ribosome	2.92	0.06	0.05	84K	0.979 *	3.167
rpoB	RNA polymerase	1.49	0.62	0.00	446I	0.965 *	1.473
rpoC1	RNA polymerase	1.30	1.39	0.00	562W	0.991 **	1.294
					568P	0.964 *	1.472
					569K	0.973 *	1.275
rpoC2	RNA polymerase	2.75	0.65	0.00	734S	0.990 *	2.728
					735K	0.980 *	2.706
rps2	subunit of ribosome	1.39	0.82	0.00	67G	0.998 **	1.384
					129F	0.954 *	1.332
					130Q	0.960 *	1.339
					131S	0.959 *	1.337
					235S	0.991 **	1.376
rps4	subunit of ribosome	3.81	0.68	0.00	25K	0.983 *	4.044
					99A	0.956 *	3.661
rps8	subunit of ribosome	1.54	1.11	0.02	55N	0.969 *	1.502
					93Q	0.996 **	1.537
ycf2	Other genes	3.31	0.05	0.00	3G	0.986 *	2.481
					117S	0.992 **	2.491
					120S	0.963 *	2.444
					429R	0.958 *	2.435
					484Q	0.962 *	2.441
					627V	0.997 **	2.498
					692K	0.984 *	2.478
					693T	0.966 *	2.448
					716T	0.960 *	2.439
					1040L	0.976 *	2.465
					1528S	0.982 *	2.474

LRT = likelihood ratio tests; Pr(Ka/Ks > 1) = the probability of a site to have Ka/Ks > 1 with a significant level of 0.05 * or 0.01 **. Ka: non-synonymous; Ks: synonymous.

**Table 3:** RNA edited sites in the chloroplast genome of *V. mungo*.

Tissue	Gene	Position on Gene	Base Change	Amino Acid Change	Editing Efficiency *	Codon Position	Coverage
Leaf	ndhC	323	C -> T	S -> L	0.89	2	27
Leaf	rps14	80	C -> T	S -> L	0.9	2	20
Leaf	rpoB	551	C -> T	S -> L	0.53	2	19
Leaf	rpoB	566	C -> T	S -> L	0.61	2	18
Leaf	rpoC1	41	C -> T	S -> L	0.75	2	16
Leaf	rps2	134	C -> T	T -> I	0.95	2	64
Shoot	rps2	134	C -> T	T -> I	0.95	2	21
Root	rps2	134	C -> T	T -> I	0.93	2	15
Leaf	rps2	248	C -> T	S -> L	0.81	2	16
Leaf	atpF	92	C -> T	P -> L	0.92	2	38
Leaf	psaI	79	C -> T	H -> Y	0.78	1	54
Leaf	psbF	44	C -> T	S -> F	0.7	2	37
Leaf	psbE	124	C -> T	P -> L	0.92	1	34
Shoot	psbE	124	C -> T	P -> L	0.88	1	21
Leaf	petL	5	C -> T	P -> L	0.43	2	20
Leaf	rps18	221	C -> T	S -> L	0.67	2	26
Leaf	clpP	2041	C -> T	H -> Y	0.82	1	16
Shoot	clpP	2041	C -> T	H -> Y	0.71	1	23
Flower	clpP	2041	C -> T	H -> Y	0.74	1	36
Pod	clpP	2041	C -> T	H -> Y	0.39	1	100
Root	clpP	2041	C -> T	H -> Y	0.66	1	69
Leaf	psbN	104	C -> T	S -> F	0.31	2	78
Flower	psbN	104	C -> T	S -> F	0.47	2	54
Leaf	rpoA	803	C -> T	S -> L	0.32	2	32
Leaf	ndhD	620	C -> T	S -> L	0.28	2	61
Leaf	ndhD	824	C -> T	S -> L	0.35	2	15
Leaf	ndhD	1115	C -> T	T -> I	0.67	2	31
Leaf	ndhE	74	C -> T	P -> L	0.95	2	18
Leaf	ndhA	20	C -> T	S -> F	0.85	2	40
Leaf	ndhA	341	C -> T	S -> L	0.82	2	42
Shoot	ndhA	341	C -> T	S -> L	0.48	2	22
Leaf	ycf4	20	G -> A	R -> K	0.14	2	55
Leaf	ycf4	309	G -> A	K -> K	0.24	3	40
Leaf	ycf4	316	G -> A	E -> K	0.24	1	21

* Editing efficiencies were calculated as a ratio of reads with the edited site to total reads mapped to that locus.

## Supplementary Materials

The following are available online at https://www.mdpi.com/2223-7747/9/9/1247/s1, Figure S1: Read coverage of the assembled genome, Figure S2: Phylogenetic tree of 69 plant species, Figure S3: Edited sites on *ycf4*, Table S1: Orthologous genes calculated from the chloroplast genome of twelve legume species with OrthoFinder program.
