# Supplementary material for: The Genome and Transcriptome Analysis of the Vigna mungo Chloroplast
Source: Plants (Basel). 2020 Sep 21;9(9):1247. doi: 10.3390/plants9091247 (PMC7570002; doi:10.3390/plants9091247)
Supplement: Supplementary file 1 [file plants-09-01247-s001.pdf]

Supplementary figures and tables

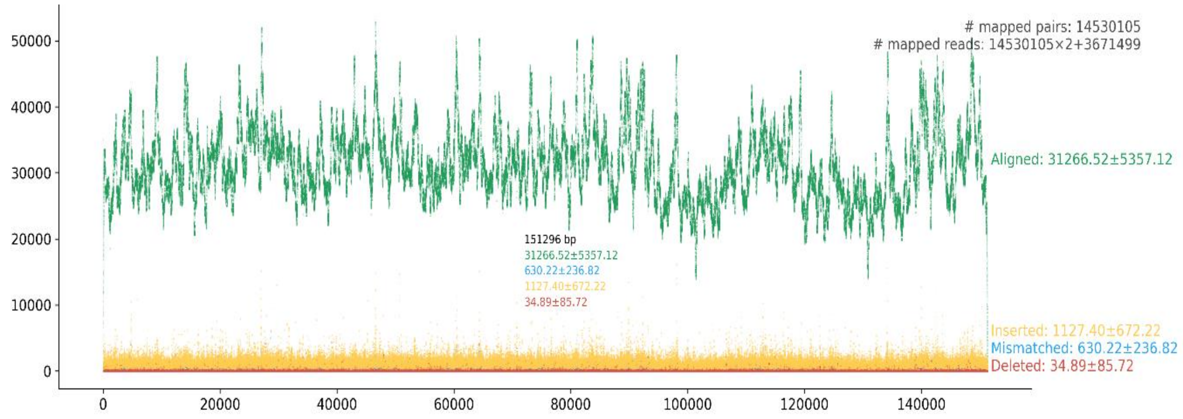

Figure S1 Read coverage of the assembled genome. The paired-end (PE) reads were remapped to the assembled genome. The number aligned bases and the polymorphic sites were counted from the mapping result.

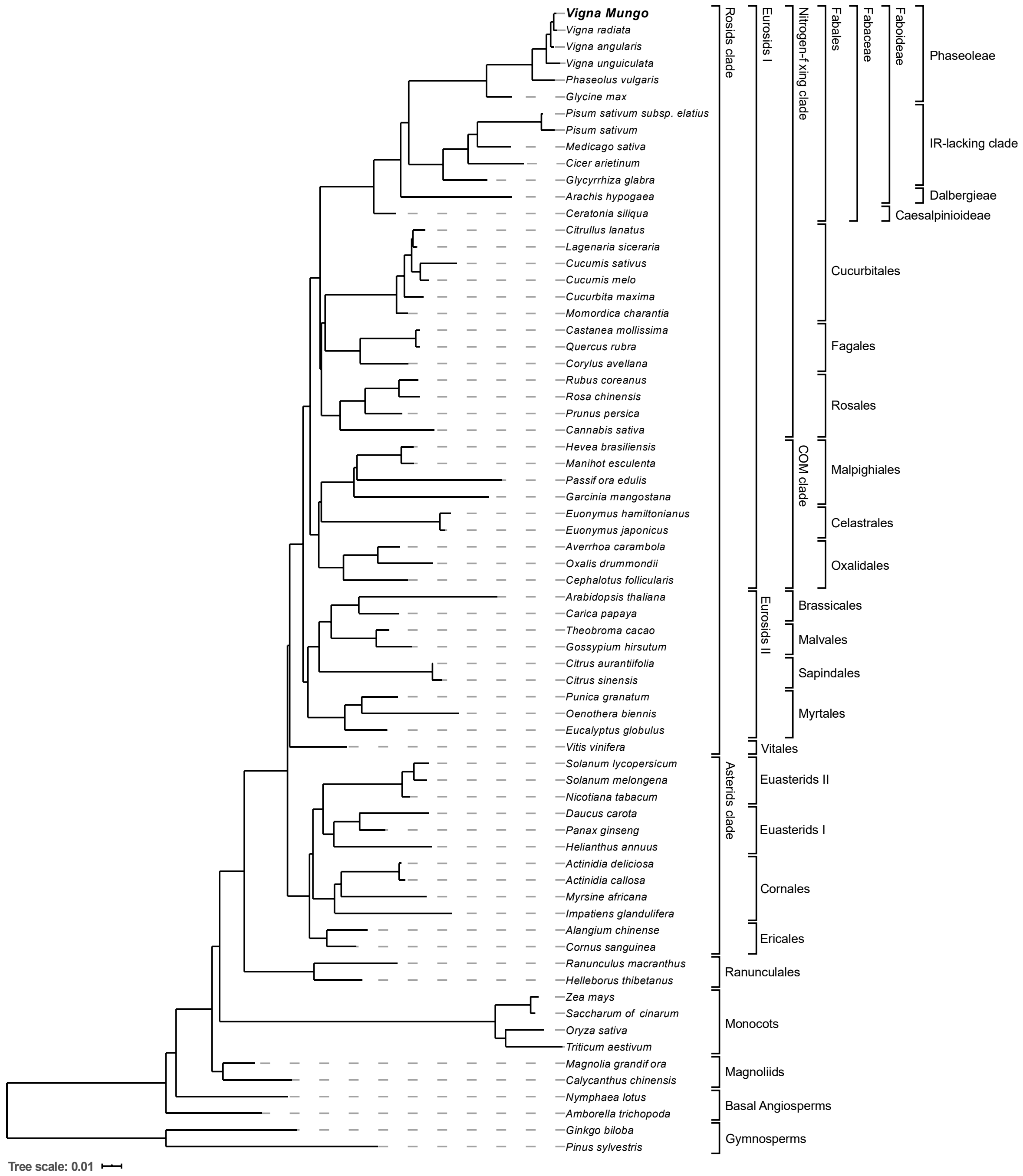

Figure S2 Phylogenetic tree of 69 plant species. The tree was calculated from the connected sequence of the 38 orthologous proteins.

```
P.vulgaris      ATGAGTATATTGAAAAAACGATCAAAAGAAGTACTGATATATTCTATAACAGAGT---CA   57
V.angularis     ATGAATATTTTTAAAAAAAGATCAAAAGAAGTATTGATCTATTCTATAACAGGGTCTCGA   60
V.Mungo         ATGAATATTTTAAAAAAAAGATCAAAAGAAGTACTGATCTATTCTATAACAGGGTCTCGA   60
V.radiata       ATGAATATTTTAAAAAAAAGATCAAAAGAAGTACTGATCTATTCTATAACAGGGTCTCGA   60
                **** *** ** ****** ************** **** ************* **    *

P.vulgaris      AAAATAAGCAATATCTTTTCAGCCTTGATCATTTTTTTAGGTTCATTGGGGTTATTGTTG   117
V.angularis     AAAATAAGCAATATCTTTTCAGCCTTTATCATTTTGTTAGGTTCTTTGGGGTTATTGTTG   120
V.Mungo         AAAATAAGCAATATCTTTTCAGCCTTTATCATTTTGTTAGGTTCTTTGGGGTTATTGTTG   120
V.radiata       AAAATAAGCAATATCTTTTCAGCCTTTATCATTTTTTTAGGTTCTTTGGGGTTATTGTTG   120
                ************************** ******** ******** ***************

P.vulgaris      GTTGCAATTTCCAGTTATCTCGGTATGGATCTTTTCCTTTTTTCTGAGGAAATTAGTAAT   177
V.angularis     GTTGGAATTTCCAGTTATCTCGGTATGGATCTTTTCCTTTTTTCTGAGGAAATTAGTAAA   180
V.Mungo         GTTGCAATTTCCAGTTATCTCGGTATGGATCTTTTCCTTTTTTCTGAGGAAATTAGTAAT   180
V.radiata       GTTGCAATTTCCAGTTATCTCGGTATGGATCTTTTCCTTTTTTCTGAGGAAATTAGTAAT   180
                **** ******************************************************

P.vulgaris      TTCCCATTTATTCCACAAGGGGCCACGATGGCTTTTTATGGAATTGGGGGTCTCTTTATT   237
V.angularis     TTTCCATTTATTCCACAAGGGGCCACGATGGCTTTTTATGGAATTGGGGGTCTCTTTATT   240
V.Mungo         TTTCCATTTATTCCACAAGGGGCCACGATGGCTTTTTATGGAATTGGGGGTCTCTTTATT   240
V.radiata       TTTCCATTTATTCCACAAGGGGCCACGATGGCTTTTTATGGAATTGGGGGTCTCTTTATT   240
                ** *********************************************************

P.vulgaris      AGTTTTTATTTGTGGTGGATTCTTTTGTGGAATATAGGTGGTGGTTTTGATATCTTCGAT   297
V.angularis     AGTTTTTATTTGTGGTGGATTATTTTGTGGGATATAGGCGGTGGTTTTGATATCTTCGAT   300
V.Mungo         AGTTTTTATTTGTGGTGGATTATTTTGTGGGATATAGGCGGTGGTTTTGATATCTTCGAT   300
V.radiata       AGTTTTTATTTGTGGTGGATTATTTTGTGGGATATAGGCGGTGGTTTTGATATCTTCGAT   300
                ********************* ******** ******* *********************

P.vulgaris      AAAAAAAATA---AAAAAGTATGTTTTATTCGTTGGGGATTTCCTGGAAAAAATCGTCGT   354
V.angularis     AAAAAAAAGAAAAAAGAAGTATGTTTTCTTCGTTGGGGATTTCCTGGAAAAAATCGTCGT   360
V.Mungo         AAAAAAAAGAAAAAAGAAGTATGTTTTCTTCGTTGGGGATTTCCTGGAAAAAATCGTCGT   360
V.radiata       AAAAAAAAGAAAAAAGAAGTATGTTTTCTTCGTTGGGGATTTCCTGGAAAAAATCGTCGT   360
                ******** *   ** *********** ********************************

P.vulgaris      ATTATTCTCAAAATACCTATGAACGAGATTCAATCTATCAGAATAATAGCAGGAGTTCAA   414
V.angularis     ATTATTAAAAAAATACCTATGAACGATATTCAATCTATCAGAATAATAACAGGAGTTCAA   420
V.Mungo         ATTATTATAAAAATACCTATGAACGATATTCAATCTATCAGAATAATAACAGGAGTTCAA   420
V.radiata       ATTCTTATAAAAATACCTATGAACGATATTCAATCTATCAGAATAATAACAGGAGTTCAA   420
                *** **   ***************** ********************* ***********

P.vulgaris      GAACGAGGTATTTTGACTCGTACCCTTACTTATGAGAGTATCGTTTATATGGAAACAATA   474
V.angularis     GAACGAGGTATTTTTACTCGTACTCTTACTTATGAGAGTATCGTTTATATGGAAACAATA   480
V.Mungo         GAACGAGGTCTTTTTACTCGTACTCTTACTTATGAGAGTATCGTTTATATGGAAACAATA   480
V.radiata       GAACGAGGTATTTTTACTCGTACTCTTACTTATGAGAGTATCGTTTATATGGAAACAATA   480
                ********* **** ******** ************************************

P.vulgaris      GAACAGGGGTTTATTACCTTGACTCGTATTGAAGATAATTTGACTCCGCCAGAAATTGCA   534
V.angularis     GAACAGGGGTTTATTCCCTTGACTCGTATTGAAGATAATTTGACTCCACCAGAAATTGCA   540
V.Mungo         GAACAGGGGTTTATTCCCTTGACTCGTATTGAAGATAATTTGACTCCACCAGAAATTGCA   540
V.radiata       GAACAGGGGTTTATTCCCTTGACTCGTATTGAAGATAATTTGACTCCACCAGAAATTGCA   540
                *************** ******************************* ************

P.vulgaris      AATAAAGCTGGCGAATTAGCTTTTTTTTTAGGTGTACCACTTCTGTATTGA   585
V.angularis     AATAGAGCTGGCGAATTAGCTTTTTTTTTAGGTGTACCGCTTCTGTATTGA   591
V.Mungo         AATAGAGCTGGCGAATTAGCTTTTTTTTTAGGTGTACCGCTTCTGTATTGA   591
V.radiata       AATAGAGCTGGCGAATTAGCTTTTTTTTTAGGTGTACCGCTTCTGTATTGA   591
                **** ********************************* ************
```

Figure S3 Edited sites on *ycf4*. Sequence of *ycf4* from three *Vigna* species, which have this gene in their cp genomes, and *P. vulgaris* are aligned and three edited sites are marked with pink bars.

Table S1 Orthologous genes calculated from the chloroplast genome of twelve legume species with OrthoFinder program.

Gene	*Ceratonia siliqua*	*Arachis hypogaea*	*Glycine max*	*Phaseolus vulgaris*	*Vigna unguiculata*	*Vigna angularis*	*Vigna_Mungo*	*Vigna radiata*	*Glycyrrhiza glabra*	*Cicer arietinum*	*Medicago sativa*	*Pisum sativum*
rps12	2	2	2	2	2	2	2	2	1	1	1	1
rpl2	2	2	2	2	2	2	2	2	1	1	1	1
ycf2	2	2	2	2	2	2	2	2	1	1	1	1
rps7	2	2	2	2	2	2	2	2	1	1	1	1
rpl23	2	2	2	2	2	2	2	2	1	1	1	0
ndhB	2	0	2	2	2	2	2	2	1	1	1	1
rps19	1	1	1	2	2	2	2	2	1	1	1	1
ycf1	1	1	2	2	1	1	1	1	1	1	1	1
psbA	1	1	1	1	1	1	1	1	1	1	1	1
matK	1	1	1	1	1	1	1	1	1	1	1	1
rbcL	1	1	1	1	1	1	1	1	1	1	1	1
atpB	1	1	1	1	1	1	1	1	1	1	1	1
atpE	1	1	1	1	1	1	1	1	1	1	1	1
ndhC	1	1	1	1	1	1	1	1	1	1	1	1
ndhJ	1	1	1	1	1	1	1	1	1	1	1	1
rps4	1	1	1	1	1	1	1	1	1	1	1	1
ycf3	1	1	1	1	1	1	1	1	1	1	1	1
rps14	1	1	1	1	1	1	1	1	1	1	1	1
psbZ	1	1	1	1	1	1	1	1	1	1	1	1
psbC	1	1	1	1	1	1	1	1	1	1	1	1
psbD	1	1	1	1	1	1	1	1	1	1	1	1
psbM	1	1	1	1	1	1	1	1	1	1	1	1
petN	1	1	1	1	1	1	1	1	1	1	1	1
rpoB	1	1	1	1	1	1	1	1	1	1	1	1
rpoC1	1	1	1	1	1	1	1	1	1	1	1	1
rpoC2	1	1	1	1	1	1	1	1	1	1	1	1
rps2	1	1	1	1	1	1	1	1	1	1	1	1
atpI	1	1	1	1	1	1	1	1	1	1	1	1
atpH	1	1	1	1	1	1	1	1	1	1	1	1
atpF	1	1	1	1	1	1	1	1	1	1	1	1
atpA	1	1	1	1	1	1	1	1	1	1	1	1
psbI	1	1	1	1	1	1	1	1	1	1	1	1
psbK	1	1	1	1	1	1	1	1	1	1	1	1
accD	1	1	1	1	1	1	1	1	1	1	1	1
psaI	1	1	1	1	1	1	1	1	1	1	1	1
cemA	1	1	1	1	1	1	1	1	1	1	1	1
petA	1	1	1	1	1	1	1	1	1	1	1	1
psbJ	1	1	1	1	1	1	1	1	1	1	1	1
psbL	1	1	1	1	1	1	1	1	1	1	1	1
psbF	1	1	1	1	1	1	1	1	1	1	1	1
psbE	1	1	1	1	1	1	1	1	1	1	1	1

petL	1	1	1	1	1	1	1	1	1	1	1	1
petG	1	1	1	1	1	1	1	1	1	1	1	1
psaA	1	1	1	1	1	1	1	1	1	1	1	1
psaB	1	1	1	1	1	1	1	1	1	1	1	1
psaJ	1	1	1	1	1	1	1	1	1	1	1	1
rps18	1	1	1	1	1	1	1	1	1	1	1	1
rpl20	1	1	1	1	1	1	1	1	1	1	1	1
clpP	1	1	1	1	1	1	1	1	1	1	1	1
psbB	1	1	1	1	1	1	1	1	1	1	1	1
psbT	1	1	1	1	1	1	1	1	1	1	1	1
psbN	1	1	1	1	1	1	1	1	1	1	1	1
psbH	1	1	1	1	1	1	1	1	1	1	1	1
petB	1	1	1	1	1	1	1	1	1	1	1	1
petD	1	1	1	1	1	1	1	1	1	1	1	1
rpoA	1	1	1	1	1	1	1	1	1	1	1	1
rps11	1	1	1	1	1	1	1	1	1	1	1	1
rpl36	1	1	1	1	1	1	1	1	1	1	1	1
rps8	1	1	1	1	1	1	1	1	1	1	1	1
rpl14	1	1	1	1	1	1	1	1	1	1	1	1
rpl16	1	1	1	1	1	1	1	1	1	1	1	1
rps3	1	1	1	1	1	1	1	1	1	1	1	1
rps15	1	1	1	1	1	1	1	1	1	1	1	1
ndhH	1	1	1	1	1	1	1	1	1	1	1	1
ndhA	1	1	1	1	1	1	1	1	1	1	1	1
ndhI	1	1	1	1	1	1	1	1	1	1	1	1
ndhG	1	1	1	1	1	1	1	1	1	1	1	1
ndhE	1	1	1	1	1	1	1	1	1	1	1	1
psaC	1	1	1	1	1	1	1	1	1	1	1	1
ccsA	1	1	1	1	1	1	1	1	1	1	1	1
rpl32	1	1	1	1	1	1	1	1	1	1	1	1
ndhF	1	1	1	1	1	1	1	1	1	1	1	1
ndhK	1	0	1	1	1	1	1	1	1	1	1	1
ndhD	1	1	1	1	1	0	1	1	1	1	1	1
ycf4	1	1	0	1	0	1	1	1	1	0	1	0
rpl33	1	1	1	0	0	0	0	0	1	1	1	1
rps16	1	0	1	0	0	0	0	0	0	0	0	0
ycf68	0	2	0	0	0	0	0	0	0	0	0	0
